# Effects of secretome on cisplatin-induced testicular dysfunction in rats

**DOI:** 10.14202/vetworld.2018.1349-1356

**Published:** 2018-09-29

**Authors:** Surya Agus Prihatno, Irma Padeta, Arinda Devi Larasati, Betty Sundari, Annisa Hidayati, Yuda Heru Fibrianto, Teguh Budipitojo

**Affiliations:** 1 Department of Reproduction and Obstetrics, Faculty of Veterinary Medicine, Universitas Gadjah Mada, Yogyakarta, 55821, Indonesia; 2 Department of Anatomy, Faculty of Veterinary Medicine, Universitas Gadjah Mada, Yogyakarta, 55821, Indonesia; 3 Department of Physiology, Faculty of Veterinary Medicine, Universitas Gadjah Mada, Yogyakarta, 55821, Indonesia

**Keywords:** cisplatin, cytokeratin, secretome, spermatogenesis, testicular dysfunction, vimentin

## Abstract

**Background:**

Testicular dysfunction is a degenerative disorder characterized by failure in the synthesis of reproductive hormones and spermatogenesis. Secretome derived from the human umbilical mesenchymal stem cell (MSC) has been reported to repair some degenerative disorders.

**Aim:**

This study aimed to investigate the effect of secretome derived from the human umbilical MSCs on cisplatin-induced testicular dysfunction in rats.

**Materials and Methods:**

Thirty-six male Wistar rats were divided into the control and secretome-treated groups. In the secretome-treated group, testicular dysfunction was induced by 3 mg/kg BW of cisplatin intraperitoneally 3 times with 3-day intervals. The secretome-treated group was divided according to dose: Low-dose (0.2 mL/kg BW) and high-dose (0.5 mL/kg BW) groups. Secretomes were injected intraperitoneally once a week for 3 weeks. 1 week after the injection of secretome, the cauda epididymis of the rats was removed for spermatozoa evaluation and histological examination.

**Result:**

After the injection of secretome, the sperm motility of the high-dose group showed thin wave-like, rare, and slow movements. No abnormal sperm morphology was observed in all the treated groups. The number of spermatozoa increased gradually in the high-dose group after the injection of secretome. The developmental stages of the spermatogenic cells were complete in both spermatozoa groups after the injection of secretome. However, the spermatozoa in the seminiferous tubules of the high-dose group were denser. Vimentin and cytokeratin immunoreactivities were very strong in the high-dose group 1 week after the second secretome injection.

**Conclusion:**

High-dose secretome derived from the human fetal umbilical cord could increase the number and motility of sperms in rats with cisplatin-induced testicular dysfunction. The administration of high-dose secretome was effective 1 week after the second dose, as indicated by very strong immunoreactivity for vimentin and cytokeratin. Moreover, secretome could promote the regeneration of the seminiferous tubules of both the groups.

## Introduction

Secretome is a factor found in the stem cell culture medium. This factor may repair the tissues of the organs that were damaged by various degenerative disorders [1]. A previous study has reported that secretome derived from the fetal human umbilical cord mesenchymal stem cell (HUC-MSC) may be an effective regenerative agent for β-cell pancreatic regeneration in Type 1 diabetes mellitus [2] and for skin regeneration in incisional [3] and burn wound healing [4]. The administration of cisplatin as a chemotherapeutic agent [5] is limited because it causes side effects, such as reproductive toxicity [6,7]. Moreover, it could decrease the number and motility of spermatozoa and increase abnormal spermatozoa morphology in patients with neoplastic disorders [8,9].

Reproductive toxicity is caused by the suppression of steroidogenesis and the generation of free radicals, and then, it progresses to testicular dysfunction [10]. As a degenerative disorder, testicular dysfunction is characterized by failure in the synthesis of reproductive hormones and spermatogenesis. Vimentin and cytokeratin are intermediate filaments that support the structural formation and functioning of the spermatogenic cells, Sertoli, and Leydig cells. Cisplatin-induced testicular cell damage would promote vimentin and cytokeratin localization in the spermatogenic, Sertoli, and Leydig cells.

Studies on the use of secretome in promoting the recovery of various degenerative disorders and its effects on the spermatogenesis of rats with cisplatin-induced testicular dysfunction have not been conducted. Therefore, we aimed to investigate the effect of secretome derived from HUC-MSC on cisplatin-induced testicular dysfunction in rats.

## Materials and Methods

### Ethical approval

This study was approved by the Ethical Committee of the Universitas Gadjah Mada with the reference number 00035/04/LPPT/V/2017.

### Experimental animal

Thirty-six male Wistar rats were adapted for 7 days before the administration of cisplatin, and these rats were fed with basal food and water *ad libitum*. Moreover, they were maintained at 12-h light-dark cycle and were divided into the control and secretome-treated groups. Testicular dysfunction was induced by cisplatin at the dose of 3 mg/kg BW [11] intraperitoneally 3 times with 3 days’ interval. Cisplatin was not administered in the control group. Then, the secretome-treated group was further divided into the low- and high-dose secretome-treated group. Secretomes were injected intraperitoneally once a week for 4 weeks at a dose of 0.2 and 0.5 mL/kg BW in the low- and high-dose secretome-treated groups, respectively. The rats were sacrificed every 1 week after the injection of secretome, and the cauda epididymis of the rat was collected for spermatozoa examination. Then, the testicles were collected for histological examination.

### Spermatozoa examination

Sperm motility and morphology were evaluated after the cauda epididymis was immediately collected. The cauda epididymis was sliced in the Petri dish and diluted in saline solution at 37°C. Sperm motility was assessed using the light microscope at 500× and was then graded according to a mass movement, as shown in Table-1. After the assessment of sperm motility, sperm morphology was immediately evaluated using the light microscope at 500×. The number of sperm was measured using the Neubauer chamber and was counted as millions/mL. The results were descriptively and quantitatively analyzed.

**Table-1:** Mass movement grade of spermatozoa.

Grade	Interpretation
+++	Dense, cloudy, and fast moving
++	Thin waves and rare and slow movements
+	Individual progressive movement

### Hematoxylin and eosin (H&E) staining

Testicular morphology was examined using the H&E method. The tissue slides were deparaffinized in xylene and rehydrated in gradual concentrations of ethanol for 5 min. Then, they were incubated in Harris hematoxylin solution for 10 min at room temperature and rinsed in running water for 10 min before incubation in eosin solution for 20 min at room temperature. Finally, they were dehydrated in gradual concentrations of ethanol for 3 min, cleared in xylene for 5 min, and mounted. The nucleus was visualized in purple color, and the cytoplasms were visualized in red color. The stained tissue slides were assessed using the light microscope with Optilab^®^ camera. The morphology and structure of the spermatogenic cells in the seminiferous tubules were observed and analyzed descriptively.

### Vimentin and cytokeratin immunohistochemical staining

Vimentin and cytokeratin immunolocalizations in the testicular tissues were visualized using the streptavidin-biotin complex method (Starr Trek Universal HRP Detection System Kit, Biocare Medical, USA). The tissue slides were deparaffinized and dehydrated and were then rinsed in running water for 10 min. The tissue slides were incubated in blocking endogenous peroxidase solution for 20 min at room temperature and then rinsed with phosphate buffer saline (PBS) 3 times for 5 min. The tissues were incubated in Biocare’s background sniper solution for 20 min at room temperature. Anti-vimentin and anti-cytokeratin primary antibodies (1:100) were used on the tissue slides at 4°C overnight.

The tissue slides were rinsed in PBS 3 times for 5 min and were incubated in Trekkie Universal Link solution for 20 min at room temperature. Before incubation, the tissue slides were rinsed with PBS 3 times for 5 min. Immunoreactive sites were visualized after using Betazoid DAB Chromogen solution (1:50) on the tissue slide at room temperature. Then, the tissue slides were counterstained with Harris hematoxylin and rinsed in running water for 10 min before being dehydrated, cleared, and mounted. Vimentin and cytokeratin intensities were assessed using the light microscope with Optilab^®^ camera and were analyzed semiquantitatively.

## Results

### Sperm count, motility, and morphology

**Figure-1:**
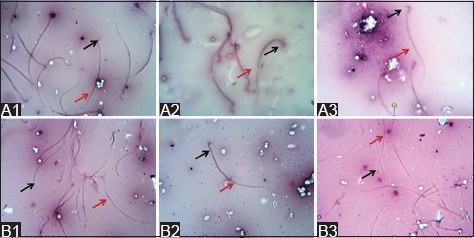
Sperm morphology of the secretome-treated group (eosin and nigrosin stain; 500× magnification). No abnormal sperm morphology was observed in the control group (1), low-dose group (2), and high-dose group (3) 1 week after the first (A) and third (B) secretome injections. The head (black arrow) and tail (red arrow) of the sperm showed the normal morphology.

The sperm motility of the low-dose group had individual progressive movement, and no changes were observed after the first, second, and third administration of secretome. However, the sperm of the high-dose group had thin waves and rare and slow movements after the second, third, and fourth administration of secretome (Table-2). No abnormal sperm morphology was observed in all the treated groups after the administration. Figure-1 shows the normal sperm morphology of the control, low-dose, and high-dose groups 1 week after the first and third administration of secretome.

**Table-2:** Sperm motility grading in the control and secretome-treated groups.

Group	1 week after the first treatment	1 week after the second treatment	1 week after the third treatment	1 week after the fourth treatment
Control	+++	+++	+++	+++
Low dose	+	+	+	ND
High dose	+	++	++	++

ND=No data

Table-3 shows that the number of spermatozoa decreased 1 week after the second secretome injection and then increased 1 week after the third secretome injection in the low-dose group. By contrast, the number of spermatozoa gradually increased 1 week after the first administration of secretome until the last secretome injection in the high-dose group. The number of spermatozoa in the high-dose group was higher than that in the control group 1 week after the third administration of secretome.

**Table-3:** Number of spermatozoa per ml in the control and secretome-treated groups.

Group	1 week after the first treatment	1 week after the second treatment	1 week after the third treatment	1 week after the fourth treatment
Control	180×10^6^	214×10^6^	181×10^6^	315×10^6^
Low dose	47×10^6^	21×10^6^	34×10^6^	ND
High dose	72×10^6^	190×10^6^	260×10^6^	307×10^6^

ND=No data

### Morphology of the spermatogenic cells in the seminiferous tubules

**Figure-2:**
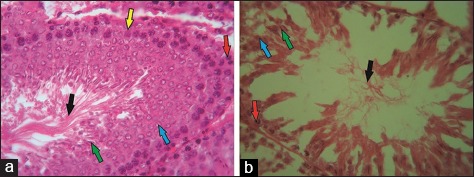
Normal (a) and cisplatin-induced seminiferous tubules (b) (H & E, 500×). Spermatogonia (red arrow), primary spermatocytes and spermatids (blue and green arrow), spermatozoa (black arrow), and Sertoli cells (yellow arrow) were observed in the normal seminiferous tubules. Conversely, severe damage was observed in the cisplatin-induced seminiferous tubules.

Figure-2 shows the morphology of the normal spermatogenic cells in the seminiferous tubules of the control group. The complete stage of spermatogenic cells and spermatozoa was observed in the lumen of the normal seminiferous tubules. The cisplatin-induced group had damaged seminiferous tubules, as indicated by the incomplete development stage of spermatogenic cells. The loss of spermatogonia and Sertoli cells was observed in the basal membrane of the seminiferous tubules.

**Figure-3:**
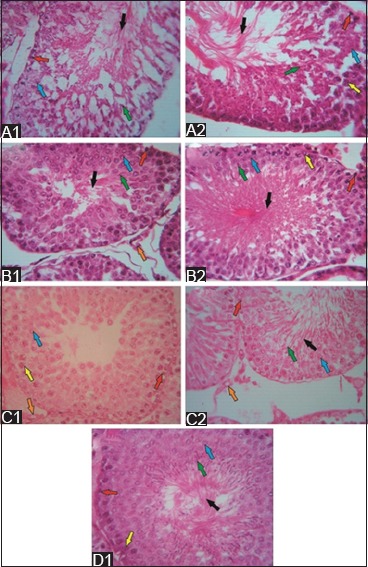
The structure of seminiferous tubules after secretome injection in the low- and high-dose groups (hematoxylin and eosin staining; 500×). 1 week after secretome injection, few spermatogonia (red arrow) and spermatozoa (black arrow) were observed in the low-dose group (A1), and few primary spermatocytes (blue arrow) were observed in the high-dose group (A2). The regenerative effects were observed in both groups 1 week after the second secretome injection. That is, the spermatogenic cells were complete. However, spermatozoa were denser in the seminiferous tubules (B2) of the high-dose group than in those of the low-dose group (B1). 1 week after the third secretome injection, low- and high-dose group (C1 and C2) showed complete spermatogenic cells. However, no spermatozoa were noted in the low-dose group. 1 week after the fourth secretome injection in the high-dose group (D1), the complete stage of spermatogenic cells and spermatozoa was observed in the lumen of the seminiferous tubules.

One week after the first administration of secretome, the low-dose group had incomplete spermatogenic cells, as indicated by few spermatogonia and spermatozoa in the basal membrane and lumen of the seminiferous tubules, respectively (Figure-3A1). The seminiferous tubules of the high-dose group were still damaged. However, the spermatogenic cells were denser than that of the low-dose group. The number of spermatogenic cells in the first stage of spermatogenesis (primary spermatocytes) was low in the high-dose group. However, the spermatogonia were complete, and several spermatozoa were observed in the lumen of the seminiferous tubules (Figure-3A2).

One week after the second administration of secretome, tissue repair was observed in the low- and high-dose groups, as indicated by the structural improvement in the seminiferous tubules. Spermatogonia were observed in the basal membrane. Furthermore, the presence of primary spermatocytes and Leydig cells was observed in both the groups. However, spermatozoa were denser in the lumen of the seminiferous tubules of the high-dose group than those of the low-dose group (Figure-3B1 and B2). The spermatogenic cells of the low- and high-dose groups completely developed 1 week after the third administration of secretome. However, no spermatozoa were observed in the seminiferous tubules of the low-dose group (Figure-3C1 and C2). 1 week after the fourth secretome injection, the spermatogenic cells and spermatozoa in the lumen of the seminiferous tubules of the high-dose group completely developed (Figure-3D1). Data about the effects of the last injection in the low-dose group were not obtained.

### Vimentin and cytokeratin immunolocalization in the seminiferous tubules

**Figure-4:**
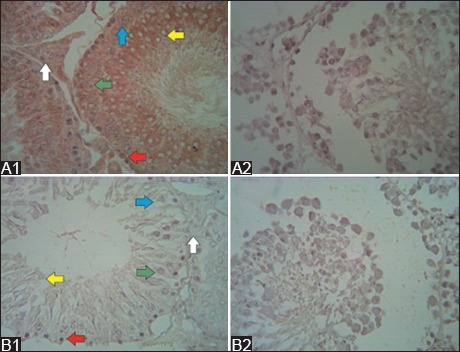
Vimentin and cytokeratin immunoreactivity in the normal and cisplatin-induced seminiferous tubules (immunohistochemistry; 500×). Very strong vimentin immunoreactivity was found in the normal spermatogenic cells (A1), and cytokeratin immunoreactivity was observed in several spermatogenic cells (B1). Moreover, no vimentin immunoreactivity (A2) and cytokeratin immunoreactivity (B2) were observed after cisplatin administration.

Vimentin immunoreactivity in the normal seminiferous tubules was very strong, particularly in the primary spermatocytes and spermatozoa (Figure-4A1). Meanwhile, cytokeratin immunoreactivity was observed in the normal seminiferous tubules. However, localization was observed in several spermatogenic cells (Figure-4B1). After the administration of cisplatin, vimentin and cytokeratin immunoreactivities were not observed. In addition, the structures of the seminiferous tubules were damaged (Figure-4A2 and B2).

**Figure-5:**
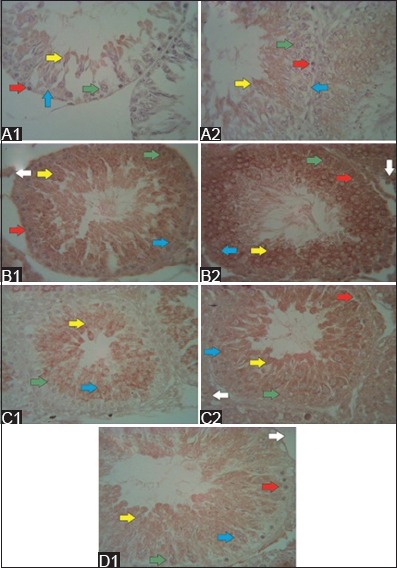
Vimentin immunoreactivity after secretome injection (immunohistochemistry; 500× magnification). In the low-dose group (A1), 1 week after the first secretome injection, a strong vimentin immunoreactivity was observed in the spermatozoa (yellow arrow) and Sertoli cells (blue arrow), whereas in the high-dose (A2) group, the immunoreactivity was moderate. In the low-dose group (B1), 1 week after the second secretome injection, a strong vimentin immunoreactivity was still observed in the spermatozoa and Sertoli cells. Meanwhile, in the high-dose group (B2), the immunoreactivity was very strong in the same cell. In the low-dose group (C1), 1 week after the third secretome injection, moderate vimentin immunoreactivity was observed in the spermatozoa and Sertoli cells, whereas in the high-dose group (C2), the immunoreactivity was strong in the same cells. Then, 1 week after the last injection in the high-dose group (D1), the vimentin immunoreactivity was moderate in the spermatozoa and Sertoli cells. Spermatogonia (red arrow), primary spermatocytes (green arrow), and Leydig cells (white arrow).

Based on the semiquantitative analysis, vimentin and cytokeratin intensities in the seminiferous tubules are shown in Table-4. 1 week after the first secretome injection, the vimentin immunoreactivity of the low-dose group was weak in the spermatogonia, moderate in the primary spermatocytes and strong in the spermatozoa and Sertoli cells (Figure-5A1). In the high-dose group, vimentin immunoreactivity was weak in the primary spermatocytes and moderate in the spermatid and Sertoli cells (Figure-5A2). Vimentin immunoreactivity was not observed in the Leydig cells of the two groups. 1 week after the second secretome injection in the low-dose group, vimentin immunoreactivity was weak in the spermatogonia, moderate in the primary spermatocytes and strong in the spermatozoa, Sertoli cells, and Leydig cells (Figure-5B1). Meanwhile, in the high-dose group, vimentin immunoreactivity in the spermatogonia and Leydig cells, spermatocytes, and spermatozoa and Sertoli cells was strong, moderate, and extremely strong, respectively (Figure-5B2). 1 week after the third secretome injection in the low-dose group, vimentin immunoreactivity in the primary spermatocytes and Leydig cells and spermatozoa and Sertoli cells were weak and moderate, respectively. However, vimentin immunoreactivity was not observed in the spermatogonia (Figure-5C1). In the high-dose group, vimentin immunoreactivity was weak in the spermatogonia and primary spermatocytes. It was moderate in the Leydig cells and strong in the spermatozoa and Sertoli cells (Figure-5C2). After the last secretome injection, vimentin immunoreactivity in the primary spermatocyte and Leydig cells was weak, and that in the spermatozoa and Sertoli cells was moderate. However, it was not observed in the spermatogonia (Figure-5D1).

**Table-4:** Semiquantitative analysis of vimentin and cytokeratin immunoreactivity in the low- and high-dose groups.

Spermatogenic cells	Cytoskeleton	1 week after the first secretome injection		1 week after the second secretome injection		1 week after the third secretome injection		1 week after the fourth secretome injection	
			
		Low	High	Low	High	Low	High	Low	High
Spermatogonia	Vimentin	+	+	+	+++	+	+	ND	−
	Cytokeratin	−	−	++	+	−	++	ND	++
Primary spermatocytes	Vimentin	++	+	++	++	+	+	ND	+
	Cytokeratin	−	+	++	+	+	++	ND	+
Spermatozoa	Vimentin	+++	++	+++	++++	++	+++	ND	++
	Cytokeratin	+	+	++	++	+	++	ND	+++
Sertoli cells	Vimentin	+++	++	+++	++++	++	+++	ND	++
	Cytokeratin	−	+	++	++	++	+++	ND	+++
Leydig cells	Vimentin	−	−	+++	+++	+	++	ND	+
	Cytokeratin	−	+	+++	++++	+	+++	ND	+++

−=Negative, +=Weak, ++=Moderate, +++=Strong, ++++=Very strong, ND=No data

**Figure-6:**
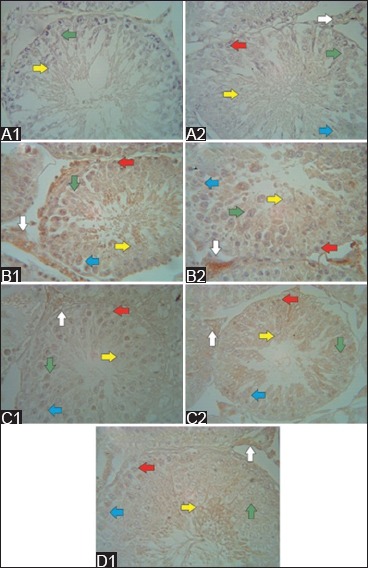
Cytokeratin immunoreactivity after secretome injection (immunohistochemistry; 500× magnification). In the low-dose group (A1), 1 week after the first secretome injection, a weak cytokeratin immunoreactivity was observed in the spermatozoa (yellow arrow), whereas in the high-dose group (A2), the immunoreactivity was weak in all spermatogenic cells, Sertoli cells (blue arrow), and Leydig cells (white arrow). In the low-dose group (B1), 1 week after the second secretome injection, a strong cytokeratin immunoreactivity was observed in the Leydig cells, whereas in the high-dose group (B2), very strong immunoreactivity was noted in the Leydig cells. In the low-dose group (C1), 1 week after the third secretome injection, moderate cytokeratin immunoreactivity was observed in the spermatozoa and Sertoli cells, whereas in the high-dose group (C2), a strong immunoreactivity was found in the Sertoli and Leydig cells. Then, 1 week after the last injection, the high-dose group (D1) showed a strong cytokeratin immunoreactivity in the spermatozoa and Sertoli and Leydig cells.

1 week after the first secretome injection in the low-dose group, cytokeratin immunoreactivity was weak in the spermatozoa and Sertoli cells (Figure-6A1). Meanwhile, in the high-dose group, cytokeratin immunoreactivity was weak in the spermatozoa, Sertoli cells, and Leydig cells (Figure-6A2). 1 week after the second secretome injection in the low-dose group, cytokeratin immunoreactivity was moderate in the spermatogonia, primary spermatocytes, spermatozoa, and Sertoli cells, and it was strong in the Leydig cells (Figure-6B1). In the high-dose group, cytokeratin immunoreactivity was weak in the spermatogonia and primary spermatocytes. Meanwhile, cytokeratin immunoreactivity was moderate in the spermatozoa and Sertoli cells and very strong in the Leydig cells (Figure-6B2). 1 week after the third secretome injection in the low-dose group, cytokeratin immunoreactivity was weak in the spermatogonia, primary spermatocytes, spermatozoa, and Leydig cells. However, it was moderate in the Sertoli cells (Figure-6C1). In the high-dose group, cytokeratin immunoreactivity was moderate in the spermatogonia and spermatozoa and strong in the Sertoli and Leydig cells (Figure-6C2). 1 week after the last secretome injection in the high-dose group, cytokeratin immunoreactivity was weak in the primary spermatocytes, moderate in the spermatogonia and strong in the spermatozoa, Sertoli cells, and Leydig cells (Figure-6D1).

## Discussion

Testicular toxicity is one of the serious side effects of cisplatin, which is used in chemotherapy [12]. Parenchymal atrophy in the seminiferous tubules had been reported after the administration of cisplatin [13], which causes loss of spermatogonia [14], abnormalities in the Leydig cells [6,15], and suppression of testosterone production [10,14]. This condition could progress to testicular dysfunction. In the present study, damage in the testicular structure was observed in rats after the administration of 3 mg/kg BW cisplatin intraperitoneally 3 times with 3 days’ interval. Cisplatin-induced testicular damage was characterized by cell apoptosis, Leydig cell dysfunction, and infertility [16]. This condition caused the inhibition of testosterone production and spermatogenesis. Cisplatin-induced testicular cell damage is caused by oxidative stress [6], which induces cellular damage by the generation of reactive oxygen species [10]. Oxidative stress could progressively damage the DNA and interfere with cell replication and transcription. Cisplatin also suppresses antioxidant enzyme activity and increases hydrogen peroxide and lipid peroxide levels in the testes and epididymis [14,17]. Severe testicular toxicity induced by cisplatin was infaust diagnosed [16]. This pathogenesis could explain the process of cell damage induced by cisplatin.

The decrease in sperm motility and number was observed in rats with testicular dysfunction induced by cisplatin. This finding showed that cisplatin deteriorated sperm quality and spermatogenesis [7,18]. However, in the present study, no abnormal sperm morphology was observed. On the contrary, the administration of cisplatin 15 times could cause various abnormalities in the head and tail of sperms [14]. The administration of cisplatin for 3 times did not cause sperm abnormalities. High-dose secretome might promote the regeneration of spermatogenic and Leydig cells. 1 week after the first, second, third, and fourth secretome injections, the number and motility of the sperms gradually increased in the high-dose group.

In the present study, the regenerative effect of secretome is characterized by the presence of spermatogenesis. Histologically, complete spermatogenic cells in the seminiferous tubules were gradually observed in the low-dose group after the 3^rd^ secretome injection and in the high-dose group after the fourth secretome injection. Spermatogenic cells could be observed and distinguished, and spermatogonia in the basal membrane would involve the primary spermatocytes in the mitotic stage. In both the groups, secondary spermatocytes were not observed due to the rapid meiotic stage of the spermatozoa in the seminiferous tubules [19]. In addition, the regeneration of Sertoli and Leydig cells was also observed. Sertoli cells play a primary role in spermatogenesis and testosterone production [20]. Spermatogenic, Sertoli, and Leydig cells were correlated to each other in terms of spermatogenesis and testosterone synthesis. Testicular dysfunction might interfere with spermatogenesis and testosterone synthesis due to cell damage. Secretome derived from the HUC-MSC can migrate, proliferate, and differentiate in the tissues or cells for regenerative process [21,22]. The regeneration of spermatogenic, Sertoli, and Leydig cells in the seminiferous tubules can be caused by growth factors and cytokines contained in the secretome.

The effects of secretome also supported the presence of cytoskeletal proteins in the spermatogenic cells after secretome injection. The following are the three major cytoskeletal proteins: Microfilament, intermediate filament, and microtubules. Vimentin and cytokeratin, which are intermediate filaments, were observed in this present study. In normal conditions, during the developmental stage, vimentin was detected in the Sertoli cells of both fetuses and adults [23-26]. The maturation of Sertoli cells was supported by the presence of vimentin in the cells. Membrane formation, which is known as the desmosome-like junction formed by vimentin, would connect the Sertoli cells with the adjacent germinal cells in the seminiferous tubules. Vimentin distribution depends on the spermatogenesis cycle, and they would be distributed along with the head of the spermatozoa. Vimentin would maintain the integrity of the spermatogenic cells and their connection [27]. Another study has reported that vimentin was also found in the Leydig cells of mature or immature testes [28]. In this present study, 1 week after the second secretome injection, vimentin immunoreactivity was strong in the spermatogonia. Vimentin immunoreactivity in primary spermatocytes, spermatozoa, Sertoli cells, and Leydig cells increased and then decreased dynamically in both the groups every 1 week after the injection. However, an extremely strong vimentin immunoreactivity was observed in the spermatozoa, Sertoli cells, and Leydig cells 1 week after the second secretome injection at high dose. Vimentin was observed in the spermatogenic, Sertoli, and Leydig cells because vimentin supported and controlled the cell structure and function of fetuses or adults [26]. Very strong vimentin immunoreactivity indicated a regenerative process.

Cytokeratin in rats was observed in the spermatogenic, Sertoli, and Leydig cells [23-26]. Cytokeratin was found in the Sertoli cells during the development stage only. However, sometimes, it was observed before the last spermatogenesis stages. Another study has reported that cytokeratin had not been observed in spermatogenic cells. However, it was noted during the pre-meiotic, meiotic, and post-meiotic stages [29]. This result was contrary to that in this study. Cytokeratin immunoreactivity in the Sertoli cells was moderate in the low-dose group 1 week after the second secretome injection. Meanwhile, it increased gradually and was strong in the high-dose group 1 week after the third secretome injection. Then, it decreased after the last injection. The dynamic increase and decrease in cytokeratin immunoreactivity were observed in the spermatogonia, primary spermatocytes, spermatozoa, and Leydig cells every 1 week after injection. However, in the Leydig cells, very strong immunoreactivity was detected in the high-dose group 1 week after the second secretome injection. Secretome that contains growth factors and cytokines, which are regenerative agents, might play a role in the formation of cytokeratin in adult rats with cisplatin-induced testicular dysfunction. The dynamic increase and decrease in the immunoreactivity of vimentin and cytokeratin might be caused by high inflammation reaction by endogenous cytokines. The use of secretome in diabetic rat could repair β-cells of the treatment which is provided earlier. However, it could not maintain the β-cells if the treatment is provided later. It was suggested that exogenous tumor necrosis factor-α and interleukin (IL) in secretome would stimulate severe inflammation reaction [30]. Severe inflammation might inhibit vimentin and cytokeratin formation. In normal conditions, Leydig cells might secrete cytokines, such as IL-1 and IL-6. Macrophages were present in the testicular interstitium, and they may stimulate Leydig cells to produce cytokines [31,32]. An *in* *vitro* study has reported that IL-1 and IL-6 also influenced the Sertoli cells [33]. However, the structures of the seminiferous tubules and cell morphology were normal.

Secretome derived from the MSC culture medium contains growth factors and cytokines [21,22]. The various growth factors and cytokines which were secreted by various stem cells in the conditioned medium [34] might play a role in cell regeneration [1]. In this study, growth factors and cytokines in secretomes derived from HUC-MSC play a role in cell regeneration through a paracrine mechanism.

## Conclusion

High-dose secretomes derived from the human fetal umbilical cord could help to increase the number and motility of sperms in rats with cisplatin-induced testicular dysfunction. Secretome injections were effective 1 week after the second secretome injection that was administered at the high dose. That is, the immunoreactivity of vimentin and cytokeratin was very strong. However, secretome could promote the regeneration of seminiferous tubules in both the groups.

## Authors’ Contributions

SAP developed the concepts and designed the experiments, evaluated the spermatozoa, and wrote the manuscript. IP processed testicle tissues for a paraffin-embedded method and visualized vimentin and cytokeratin using immunohistochemical method. ADL created testicular dysfunction in rat with cisplatin-treated rats with secretome. BS performed euthanization of rats, collected testes, fixed sample in Bouin, and evaluated the spermatozoa. AH stained the tissues structures using H & E method. YHF produced the secretome. TB developed the concepts and designed the experiments and analyzed and interpreted the data. All authors read and approved the final manuscript.
